# Impacts of Lithology and Slope Position on Arbuscular Mycorrhizal Fungi Communities in a Karst Forest Soil

**DOI:** 10.3390/jof9121133

**Published:** 2023-11-24

**Authors:** Jin Zhao, Xunyang He, Dan Xiao, Meifeng Chen, Ming Cheng, Zhongcheng Wang

**Affiliations:** 1 Forestry College, Central South University of Forestry and Technology, Changsha 410004, Chinam15874806022@163.com (M.C.); 2 Key Laboratory of Agro-Ecological Processes in Subtropical Region, Institute of Subtropical Agriculture, Chinese Academy of Sciences, Changsha 410125, China; hbhpjhn@isa.ac.cn (X.H.);; 3 Huanjiang Observation and Research Station for Karst Ecosystems, Chinese Academy of Sciences, Huanjiang 547100, China

**Keywords:** arbuscular mycorrhizal fungi, lithology, slope position, forest, karst ecosystems

## Abstract

The influence of lithology and slope position on arbuscular mycorrhizal fungi (AMF) communities has been explored in various ecosystems, but there is a limited understanding of these mechanisms in karst regions. This study focused on typical karst hills with contrasting lithologies, specifically dolomite and limestone. Additionally, three slope positions (upper, middle, and lower) were investigated within each hill in karst forest ecosystems. Total phosphorus (TP) content in the soil was higher in dolomite compared to limestone. Conversely, exchangeable calcium (Ca) was lower in dolomite than in limestone. Notably, the lithology, rather than the slope position, exerted a significant impact on AMF diversity and abundance and the presence of specific AMF taxa. Dolomite exhibited greater AMF richness and a higher Shannon index in comparison to limestone when not accounting for slope position. The AMF community composition differed between dolomite and limestone. For instance, without considering slope position, the relative abundance of *Acaulospora*, *Diversispora*, and *Paraglomus* was higher in dolomite than in limestone, while the relative abundance of *Claroideoglomus* displayed an opposing trend. Furthermore, a more complex interaction among AMF taxa was observed in dolomite as compared to limestone, as evidenced by an increase in the number of nodes and edges in the co-occurrence networks within the dolomite. The genera *Glomus*, *Claroideoglomus*, and *Diversispora* exhibited a higher number of links with each other and with other AMF taxa. The study identified TP and Ca as the primary factors determining variations in AMF diversity between dolomite and limestone. Consequently, it is imperative to consider the underlying lithology and soil conditions when addressing the restoration of degraded karst hilly areas.

## 1. Introduction

Karst landscapes, developing on carbonate bedrocks (e.g., limestone, dolomite, or marble), are widely recognized as exceptionally ecologically fragile ecosystems [1,2,3]. The karst region in China stands as one of the largest in the world [4]. Due to its distinct geological and hydrological conditions, this region is typically characterized by high bedrock exposure ratios, shallow soil layers, low stability, and limited nutrient availability [2,3]. Over the past century, human activities, including population growth and unsustainable agricultural practices, have led to severe land degradation in karst regions, resulting in a phenomenon known as karst rocky desertification [2]. In response to this issue, the Chinese government has initiated a series of ecological restoration projects aimed at rehabilitating the degraded land, particularly on the lower slopes. A common outcome in this region is the conversion of cropland into forests, a process referred to as agricultural abandonment [3,5,6]. The natural regeneration of vegetation or afforestation in karst areas is a complex undertaking due to the high heterogeneity of various habitats [2]. Previous studies have indicated that variations in soil nutrients and plant diversity are closely linked to different slope positions [7,8,9]. Additionally, lithologies have a significant impact on soil nutrient levels [10]. Therefore, differences in slope position and lithologies may interact to influence soil nutrient profiles, subsequently resulting in variations within the microbial community.

Arbuscular mycorrhizal fungi (AMF) play crucial functional roles in enhancing nutrient-use efficiency, improving soil health and structure, and modifying plant interactions [11,12]. It is estimated that the majority of terrestrial plants (approximately 80%) engage in mutualistic relationships with mycorrhizal fungi [11]. AMF provide host plants with essential nutrients like phosphorus (P) and nitrogen (N) while, in return, they obtain carbon (C) from the plants. The diversity and composition of AMF communities can be influenced by factors such as the host plant, soil nutrients, and the surrounding environment [11,13]. For instance, soil P availability can control AMF diversity, with abundant P availability potentially reducing AMF abundance and diversity [11,14]. Given that different habitats, such as slope positions and lithology types, can impact nutrient availability and plant diversity, it is imperative to elucidate the mechanisms through which these diverse habitats affect AMF diversity and community composition.

The geological composition of various lithologies, characterized by their distinct rock properties, is a pivotal factor influencing soil development and governing the flow of material inputs into the soil [15]. Soils originating from different bedrock types, such as dolomite and limestone, can indeed display significant differences in soil physical properties, nutrient content, and microbial community compositions [10]. Limestone and dolomite are two prevalent bedrock types in karst ecosystems, each possessing distinct characteristics [2,4]. Dolomites contain relatively high amounts of sparingly soluble detrital silicate minerals, which have a lower dissolution rate compared to limestones [16,17]. Consequently, the variations in the elements released from limestone and dolomite may lead to changes in soil nutrients, subsequently influencing the AMF community. For instance, many studies in the karst region have reported that bedrock type is a crucial factor influencing soil AMF communities due to variations in soil nutrients and plant diversity [18,19]. Nevertheless, there is a scarcity of research that directly compares the response of AMF abundance and diversity between limestone and dolomite, particularly under various slope positions.

Slope position plays a pivotal role in governing the heterogeneity of forest ecosystems by regulating plant communities, as well as the physical, chemical, and microbial properties of the soil [7,8,20]. Typically, slope position primarily influences above-ground plant communities and soil nutrient dynamics through factors like surface runoff and light, subsequently impacting the soil microbial community and its functional characteristics [20,21]. It has been observed that soil available nutrients generally increase from the upper slope to the foot slope or valley [7,22,23]. However, some studies have shown contrasting results, such as higher organic matter content in ridges compared to lower slope areas [24,25]. These differences may be attributed to variations in litter input and microclimate conditions, which can have a substantial impact on AMF communities. This suggests that the complexity and uncertainty associated with varying slope positions can lead to diverse effects on AMF colonization and diversity, particularly in different ecosystems. For example, in karst shrub ecosystems, AMF colonization was found to be higher in the lower slope compared to the upper slope, while the diversity of root-associated AMF showed the opposite pattern [7,26]. Despite numerous studies investigating the influence of slope position on AMF communities, the effects of lithologies, slope positions, and their interactions on AMF diversity and structure remain unclear.

Hence, in our study, we conducted an investigation of the AMF community in a representative karst forest, taking into account different slope positions (lower, middle, and upper slopes) and lithologies (limestone and dolomite). The primary objectives of our research were to assess the impact of lithologies and slope positions on AMF diversity and composition, as well as to compare the interactions among AMF taxa between limestone and dolomite.

## 2. Materials and Methods

### 2.1. Study Area

The study area, situated within the coordinates 107°51′–108°30′ E and 23°47′–24°35′ N, is located in Duan County, Hechi City, Guangxi Zhuang Autonomous Region, China. This region is a representative example of China’s typical karst geomorphological areas and experiences a subtropical monsoon climate. The average annual precipitation in this area is approximately 1726 mm, and the average annual temperature ranges from 19.6 °C to 21.6 °C. Limestone and dolomite are two significant types of bedrock widely distributed throughout the study area. The soil in this region is categorized as calcareous lithosols, having developed from limestone and dolomite, in accordance with the FAO/UNESCO soil classification system. The secondary forest selected for this study has naturally regenerated over a period of 30–50 years, following the abandonment of cropland. The dominant plant species in this secondary forest are *Sinoadina racemosa* and *Radermachera sinica*.

### 2.2. Soil Sampling

The study involved the collection of thirty soil samples, encompassing two distinct lithologies (limestone and dolomite), three slope positions (upper, middle, and lower slopes), and five replicates for each combination. Specifically, five replicate transects were established for each lithology (limestone and dolomite). Within each transect, the slope was further divided into three positions: upper slope, middle slope, and lower slope. Each slope position had a designated square plot (30 m × 30 m) for soil sampling, with the removal of the humus layer before sampling. At each plot, a total of fifteen soil cores (diameter 2.5 cm) at a depth of 0–15 cm were randomly taken following an “S” shaped transect pattern. Subsequently, the soil cores obtained from each individual plot were combined to create a composite sample. The collected soil samples were then transported to the laboratory, where rocks, animal matter, and plant residues were meticulously removed from the composite sample using a 2.0 mm mesh. Each composite sample was divided into two separate subsamples for further analysis. One of these subsamples was preserved in a refrigerator at a temperature of −80 °C, while the other subsample was subjected to air-drying in preparation for physicochemical analyses.

### 2.3. Soil Physiochemical Properties Analysis

Soil pH values were determined with a pH meter (FE20K; Mettler-Toledo, Greifensee, Switzerland), using a soil-to-water ratio of 1:2.5 [5]. Soil organic carbon (SOC) was measured through a wet oxidation process, involving a mixture of KCr_2_O_7_ and H_2_SO_4_, followed by titration with FeSO_4_. Total nitrogen (TN) was assessed using an elemental analyzer (Vario MAX CN; Elementar, Hanau, Germany). Total phosphorus (TP) was quantified using the acid digestion method. Available phosphorus (AP) was determined via the molybdenum blue method with H_2_SO_4_ + HClO_4_ solution [18]. Total potassium (TK) was analyzed using flame photometry after extraction with sodium hydroxide [7]. Exchangeable calcium (Ca) and magnesium (Mg) were extracted through compulsory exchange in ammonium acetate (1 mol L^−1^) at pH 7 and subsequently analyzed by inductively coupled plasma atomic emission spectrometry (ICP-AES; Agilent, Santa Clara, CA, USA) [6,7]. For detailed information regarding the soil physiochemical properties analysis, please refer to previous studies [5,6,7].

### 2.4. DNA Extraction and Amplicon Sequencing

DNA extraction for soil samples (0.3 g) was conducted using the FastDNA Spin kit for soil (MP Biomedicals, Santa Ana, CA, USA). The DNA extraction process adhered to the manufacturer’s protocol, and the concentration and quality of the extracted DNA were evaluated using a NanoDrop spectrophotometer (NanoDrop Technologies, Wilmington, DE, USA). The assessment was further confirmed through 1% (*w*/*v*) agarose gel electrophoresis. Following these procedures, the DNA samples were stored at −20 °C for subsequent analysis.

The 18S rRNA gene specific to soil AMF was amplified through a nested PCR approach. In the first round of amplification, the primers used were AML1 (ATCAACTTTCGATGGTAGGATAGA) and AML2 (GAACCCAAACACTTTGGTTTCC). For the second round of amplification, the primers used were AMV4.5NF (AAGCTCGTAGTTGAATTTCG) and AMDGR (CCCAACTATCCCTATTAATCAT). The PCR reaction mixture consisted of 1 μL of DNA template, 10 μL of 2× PCR ExTaq, and 0.50 μL of both forward and reverse primers, and sterile water was added to reach the final volume. In the second round of PCR amplification, the DNA template was diluted 50-fold using the product from the first-round PCR amplification. Additionally, the primers AMV4.5NF and AMV4.5NF were tagged with a 6 bp barcode sequence to differentiate between the samples. For the first round of nested PCR, the conditions were as follows: 5 min at 94 °C, followed by 35 cycles of 30 s at 94 °C, 45 s at 58 °C, 60 s at 72 °C, and a final elongation for 10 min at 72 °C. The conditions for the second round of PCR amplification were: 3 min at 94 °C, followed by 30 cycles of 45 s at 94 °C, 45 s at 60 °C, 60 s at 72 °C, and a final elongation for 10 min at 72 °C. The PCR products were sequenced using the Illumina HiSeq 2500 platform. Sequence analysis was conducted using the UPARSE pipeline in USEARCH (v.10.0.240) and R (R Development Core Team). More detailed information about the PCR reaction systems, conditions, and sequence analysis can be found in our previous studies [7,18,27].

### 2.5. Statistical Analysis

The data were initially assessed for normal distribution, and in order to improve normality, data transformation was applied as needed. Differences between different slope positions or lithologies were analyzed using Duncan’s test at a significance level of 5%. Two-way analysis of variance (ANOVA) was employed to determine the effects of slope position, lithology, and their interactions on AMF diversity and the relative abundance of AMF taxa. The distribution of AMF community composition at different slope positions and lithologies was assessed using non-metric multidimensional scaling (NMDS). To examine the relationship between environmental factors and AMF diversity, as well as the relative abundance of AMF taxa, Pearson correlation analysis was conducted. A co-occurring network analysis of AMF taxa was performed using Spearman’s correlation with an adjusted *p*-value threshold of <0.05. All statistical analyses were carried out using R version 4.02.

## 3. Results

### 3.1. Change in Soil Properties

**Figure 1:**
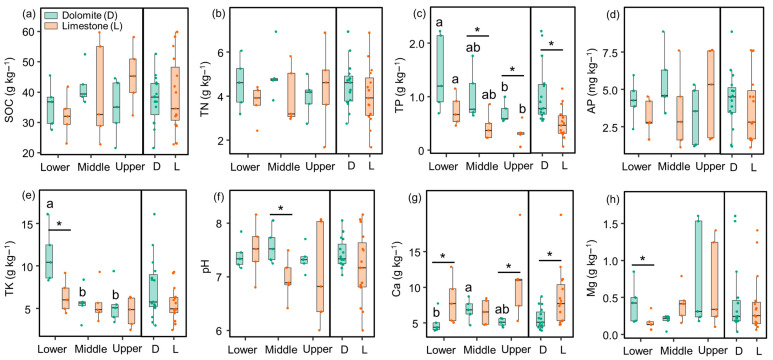
Change in soil physiochemical properties under different dolomite and limestone conditions among different slope positions. Values are means ± standard errors (*n* = 5). Different letters indicate significant differences (*p* < 0.05) among the lower, middle, and upper slopes within the same lithology. Significant differences (*p* < 0.05) between dolomite and limestone at the same slope position are indicated by asterisks. SOC, soil organic carbon; TN, total nitrogen; TP, total phosphorus; AP, available phosphorus; TK, total potassium; Ca, soil exchangeable Ca^2+^; Mg, soil exchangeable Mg^2+^. TP and Ca exhibited variations between dolomite and limestone. Additionally, TP and TK levels experienced changes across different slope positions.

The study revealed significant changes in soil properties as influenced by slope position and lithology. Specifically, TP and TK (with the exception of limestone) were found to be higher in the lower slope compared to the upper slope within the same lithology. The Ca content was lower in the lower slope compared to the middle slope in the dolomite. Regardless of slope position, TP was higher in the dolomite soil compared to the limestone soil, while Ca showed the opposite trend. However, TK was higher in the dolomite soil compared to the limestone soil, but this difference was primarily observed in the lower slope. Furthermore, SOC, TN, AP, pH, and Mg were less influenced by both slope position and lithology (Figure 1).

### 3.2. Characteristics of the Soil AMF Community

**Figure 2:**
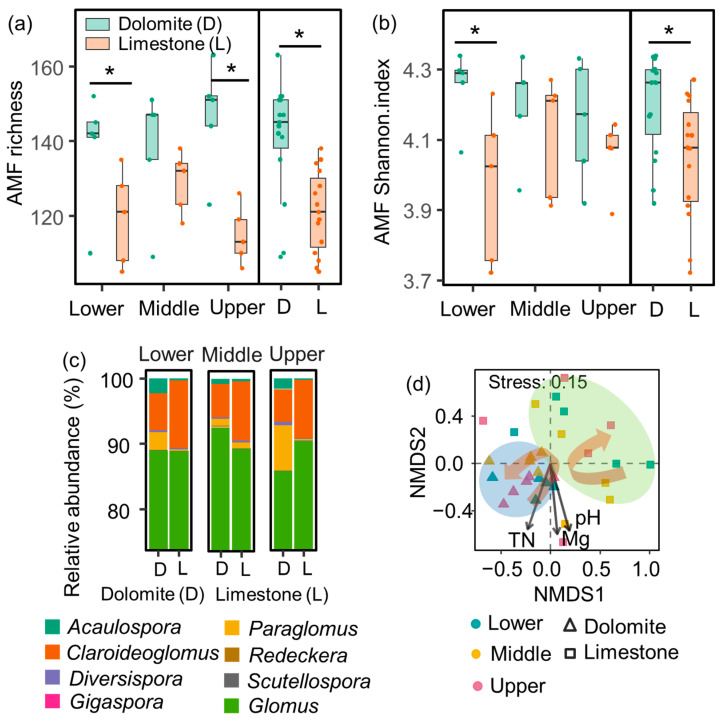
Variation in AMF diversity (**a**,**b**) and community composition (**c**,**d**). Values are means ± standard errors (*n* = 5). Significant differences (*p* < 0.05) between dolomite and limestone at the same slope position are indicated by asterisks. Vectors in (**d**) indicate statistically significant correlations between the soil properties and AMF community composition (*p* < 0.05). TN, total nitrogen; Mg, soil exchangeable Mg^2+^. The curved arrow in (**d**) illustrates the progression of AMF community composition with respect to slope position. Notably, AMF richness, Shannon index, and community composition exhibited significant differences between dolomite and limestone.

The diversity of AMF was significantly affected by lithology but remained unaffected by slope position or the interaction between slope position and lithology (Table 1). Regardless of the specific slope position, the richness and Shannon index of AMF were significantly higher in dolomite soils compared to those in limestone soils. Interestingly, the study observed a consistent level of AMF diversity across the lower, middle, and upper slope positions (Figure 2a,b).

At the genus level, the relative abundance of *Glomus* (88.8%) was the most prominent, followed by *Claroideoglomus* (7.3%), *Paraglomus* (2.5%), *Acaulospora* (1.09%), *Diversispora* (0.29%), *Scutellospora* (0.04%), and *Gigaspora* (0.01%) (Figure 2c).

**Figure 3:**
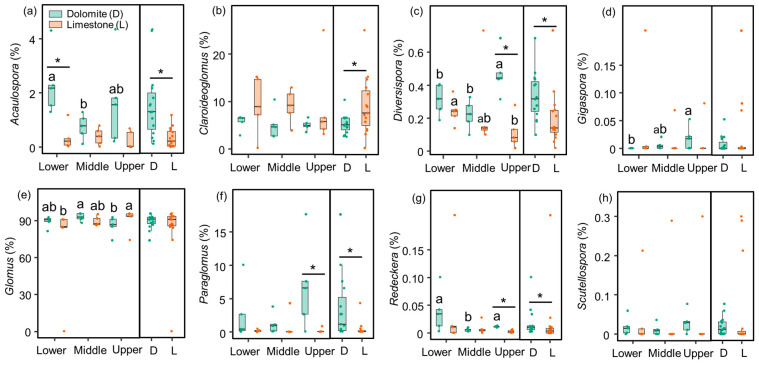
Soil AMF taxa at the genus level under different dolomite and limestone conditions among different slope positions. Values are means ± standard errors (*n* = 5). Different letters indicate significant differences (*p* < 0.05) among the lower, middle, and upper slopes within the same lithology (e.g., *Diversispora*). Significant differences (*p* < 0.05) between dolomite and limestone at the same slope position are indicated by asterisks (e.g., *Acaulospora*, *Claroideoglomus*, *Diversispora*, and *Paraglomus*).

The NMDS analysis demonstrated significant differences in the composition of the AMF community between dolomite and limestone (Figure 2d). The relative abundance of *Acaulospora*, *Claroideoglomus*, *Diversispora*, and *Paraglomus* was significantly influenced by lithology (Table 1). Regardless of the slope position, *Acaulospora*, *Diversispora*, and *Paraglomus* exhibited notably higher relative abundances in dolomite compared to limestone. In contrast, the relative abundance of *Claroideoglomus* displayed an opposite trend. Furthermore, the relative abundance of *Diversispora* was lower in the lower and middle slopes compared to the upper slope in dolomite, while the trend was reversed in limestone. Although *Gigaspora* and *Glomus* exhibited similar levels of abundance across different lithologies, their relative abundances were higher in the upper slope compared to the lower slope in dolomite and limestone, respectively (Figure 3).

### 3.3. Co-Occurrence Network Analysis among AMF Taxa under Different Lithologies

**Figure 4:**
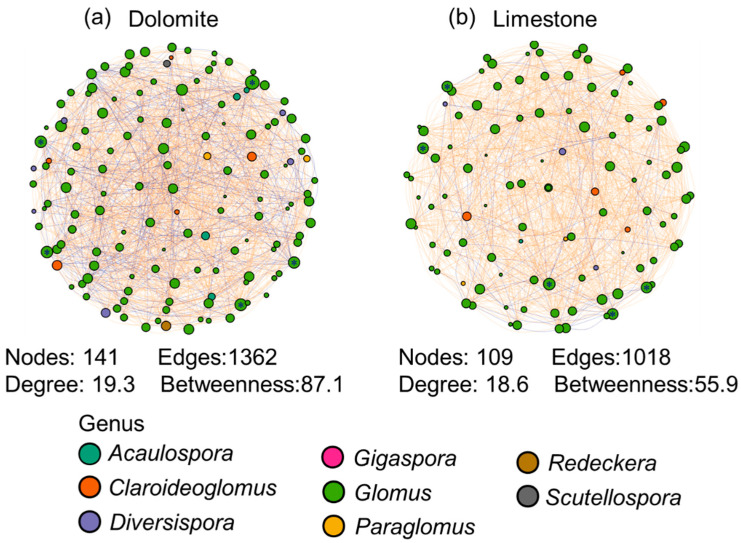
The interactions among AMF taxa at the genus level under different dolomite (**a**) and limestone (**b**) conditions based on the network analysis. The size of each node reflects its degree of connectivity, indicating the number of connections with other taxa. A larger circle size indicates more significant associations with other taxa. The orange and blue lines connecting pairs of nodes represent positive and negative interactions, respectively.

The network analysis revealed that the interactions among AMF taxa were weaker in the limestone environment compared to the dolomite environment. In the dolomite environment, the number of nodes (141 vs. 109), edges (1362 vs. 1018), mean degree (19.3 vs. 18.6), and betweenness (87.1 vs. 55.9) was significantly higher compared to the limestone environment. These findings indicate a more complex network structure within the dolomite. Additionally, the genera *Glomus*, *Claroideoglomus*, and *Diversispora* exhibited the highest number of connections among themselves and with other taxa (Figure 4).

### 3.4. The Relationships between Soil Properties and AMF Communities

**Figure 5:**
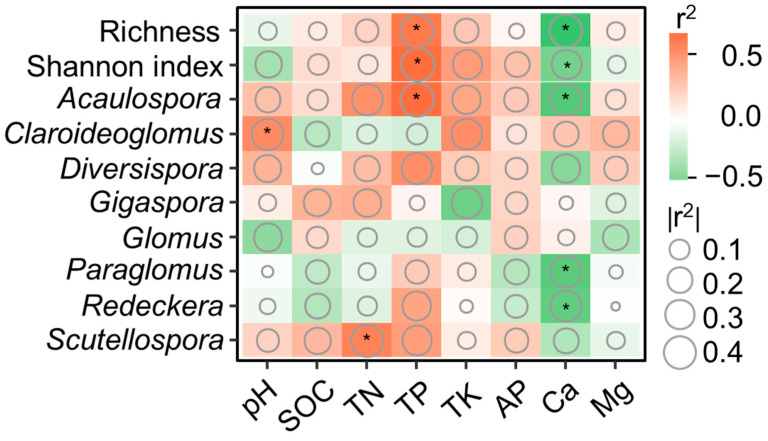
Correlation among soil properties and AMF diversity and taxa at the genus level. SOC, soil organic carbon; TN, total nitrogen; TP, total phosphorus; AP, available phosphorus; total potassium; Ca, soil exchangeable Ca^2+^; Mg, soil exchangeable Mg^2+^. TP and Ca were identified as the key factors driving variations in AMF diversity and taxa. Significant correlation was labeled in asterisks (* *p* < 0.05).

The correlation heatmap provides valuable insights into the relationships between AMF diversity, specific AMF taxa, and soil properties. Specifically, AMF richness, Shannon index, and the relative abundance of *Acaulospora* exhibit positive correlations with TP but negative correlations with Ca. In addition, the relative abundance of *Claroideoglomus* and *Scutellospora* were positively correlated with soil pH and TN, respectively. The relative abundance of *Paraglomus* and *Redeckera* is negatively correlated with Ca. Moreover, the composition of the AMF community exhibits significant correlations with soil pH, TN, and Mg (Figure 5).

## 4. Discussion

### 4.1. Effect of Slope Position and Lithology on AMF Diversity and Community Composition

The study results demonstrate that the diversity and composition of AMF communities are predominantly influenced by lithology, with a more significant impact compared to slope position or their combined effects. It was observed that soil nutrient levels, such as TP and TK, exhibited a propensity to increase from upper to lower slopes within the same lithological context. This finding agrees with previous research conducted in karst shrub ecosystems, which reported higher nutrient availability (including available N and K) and greater plant richness in the lower slopes as compared to the upper slopes [7,26]. This result is supported by several studies because of residue and sediment accumulated in the lower slope caused by soil erosion [1,9,28]. Notably, despite the elevation of TP and TK content in the lower slopes, AMF diversity and taxa showed a consistent level across different slope positions. In contrast, the intensity and abundance of AMF colonization, rather than AMF diversity, were notably elevated in the lower slopes [7]. This increased colonization intensity is attributed to the richer plant diversity observed in the lower slopes, a pattern noted in previous research conducted within karst shrub ecosystems [7]. Additionally, a previous study indicated that the diversity of root-associated AMF was greater in the upper slope compared to the lower slope [26]. These contrasting findings suggest that the diversity of soil- or root-associated AMF at different slope positions may be influenced by a combination of various factors, such as the specific sampling site and geographical distances between locations. It is worth noting that plant diversity and soil nutrient availability may also be contributing factors, although in the present study, plant diversity was not assessed in the forest ecosystems under investigation.

The influence differs between slope position and lithology, with lithology significantly affecting both AMF diversity and community composition. Regardless of the specific slope position, the dolomite exhibited higher levels of AMF diversity and Shannon index when compared to limestone. Under similar external environmental conditions, the type of bedrock, serving as the soil-forming parent material, primarily determines the composition of soil particles and mineral nutrients [29,30]. Lithology plays a crucial role in influencing soil nutrient availability by facilitating the weathering of bedrock and releasing essential elements into the soil [29,31]. Previous research conducted in the karst region has reported higher levels of soil C and N in limestone areas compared to dolomite within cropland and grassland ecosystems [5]. However, in this study, for the forest ecosystem, TP levels were higher in dolomite compared to limestone regardless of slope position. Conversely, Ca exhibited a contrasting pattern. These differing outcomes might be attributed to variations in land-use types, sampling conditions, and the specific characteristics of the study area, emphasizing the influence of these factors on soil nutrient dynamics between dolomite and limestone substrates [32]. In the present study, TP exhibited a positive correlation with AMF diversity. Consistent with our findings, numerous studies have reported that soil P is a crucial factor influencing AMF communities [27,33,34]. It is worth noting that while high soil P content tends to enhance AMF diversity [35], excess P can actually inhibit AMF growth and diversity [27]. Both dolomite and limestone are carbonate rocks with rich Ca content. In such an environment, soluble P reacts with calcium to form insoluble precipitates [36,37]. The karst forest in this study was found to be limited by P based on soil eco-enzymatic stoichiometry [37], which could explain the positive correlation between TP and AMF diversity. Additionally, the high levels of exchangeable Ca in the soil can form complexation with organic C, stabilizing SOC [38,39], which may result in lower available C content in limestone compared to dolomite. Soil C resources were identified as the key factor affecting AMF growth. This is supported by the significant negative correlation between Ca and AMF diversity in this study.

The AMF community composition significantly differed between dolomite and limestone. The relative abundance of *Diversispora* was influenced by lithology and the interaction between lithology and slope position. Additionally, the relative abundances of *Acaulospora*, *Claroideoglomus*, and *Paraglomus* were only impacted by lithology. This suggests that lithology likely plays a crucial role in shaping the diversity of AMF taxa by regulating soil nutrients and plant communities. For instance, the relative abundance of *Acaulospora* exhibited a positive correlation with TP, indicating that the higher TP content in dolomite led to greater *Acaulospora* abundance in dolomite compared to limestone. This result suggests that certain AMF taxa, such as *Acaulospora*, *Diversispora*, and *Paraglomus*, may have an advantage in dolomite with high P content. In conclusion, lithology, including dolomite and limestone, especially in karst regions, should be a primary consideration when applying AMF to enhance fragile ecosystems.

### 4.2. Stronger Interaction among AMF Taxa in the Dolomite Compared to Limestone

Co-occurrence networks were constructed using topological features based on amplicon sequence variants (ASVs) at the genus level to explore potential interactions among AMF taxa [7,18]. Microorganisms form intricate networks, participating in a variety of interactions, including both negative ones like competition and positive ones like mutually beneficial relationships [40,41]. The network analysis revealed that, in comparison to limestone, dolomite had a greater number of edges, nodes, degrees, and betweenness, indicating that the network structure in dolomite was more complex. Indeed, the higher AMF diversity in dolomite, driven by its high TP but low Ca content, suggests that increasing AMF diversity in dolomite soils could foster interactions among microbial communities, leading to stronger species co-occurrence. This finding has been demonstrated by previous studies [42,43,44]. Moreover, the ratio of negative to positive link numbers was found to be higher in the dolomite compared to limestone. Previous research in a karst region focusing on dolomite has shown that plant roots predominantly extend horizontally, which presents a challenge in accessing the moisture-retaining properties of the upper karst zone and its rich nutrient resources [45,46]. This finding supports the idea that the soil environment in the dolomite can intensify competition among different AMF taxa. Furthermore, the majority of nodes and links in the network for both dolomite and limestone were associated with the genera *Glomus*, *Claroideoglomus*, and *Diversispora*. This suggests that these keystone taxa play a crucial role in microbial networks, contributing to the maintenance of ecosystem stability and sustainability.

### 4.3. Implications for Future Management

The high heterogeneity of karst habitats is influenced by factors such as lithology and slope position, both of which play crucial roles in the restoration of vegetation [2,3,32,39]. In karst forest ecosystems, it is noteworthy that AMF diversity, abundance, and community composition are more significantly influenced by lithology rather than slope position. AMF richness and Shannon index were positively correlated with TP but negatively correlated with Ca. The differences in TP and Ca content between dolomite and limestone suggested that TP and Ca are key factors determining the variation in AMF communities across different lithologies. Although TP was higher in the lower slope compared to the upper slope in both dolomite and limestone, there was no significant difference in AMF diversity at different slope positions. In this study, we selected well-established secondary forests with relatively long recovery periods. The influence of sampling distances across varying slopes may have resulted in relatively consistent vegetation community characteristics among different slope positions, consequently leading to similar mycorrhizal diversity. Therefore, it is essential to pay attention to the varying impacts of lithology on AMF associations in the process of karst vegetation restoration.

In light of the influence of lithology on AMF diversity, it is important to note that this study did not measure the chemical properties of the bedrock. Therefore, future research should investigate the mechanisms by which bedrock chemistry affects AMF. Moreover, plant species have a significant impact on AMF communities, but we did not analyze the effect of vegetation community on AMF diversity in this study. It is necessary to explore the relationship between bedrock weathering, plant community, and AMF indices in karst ecosystems with a high degree of spatial heterogeneity in future studies.

## 5. Conclusions

In summary, this study demonstrates that AMF diversity, abundance, and community composition (e.g., *Acaulospora*, *Claroideoglomus*, *Diversispora*, and *Paraglomus*) in karst forest ecosystems are significantly affected by lithology, while the slope position showed less effect on AMF communities. This indicates that lithology, rather than slope position, is an important factor driving changes in AMF abundance and diversity in the karst forest. Greater AMF richness and Shannon index were observed in the dolomite compared to the limestone, regardless of the slope position, mainly due to variations in TP and Ca between the two lithologies. We also found stronger interactions among AMF taxa in the dolomite than in the limestone, facilitated by the higher AMF diversity in the dolomite. The genera *Glomus*, *Claroideoglomus*, and *Diversispora* were identified as keystone taxa in the network, suggesting their significant contributions to ecosystem sustainability. This study provides evidence of how AMF communities respond to lithology and slope position in the karst forest. Future research should focus on the regulation of bedrock properties in AMF communities by promoting plant growth.

## Figures and Tables

**Table 1:** Effects of lithology, slope position, and their interactions on soil physiochemical properties, AMF diversity, and AMF taxa at the genus level.

Items	Lithology	Slope Position	Lithology × Slope Position
SOC	0.21	1.47	1.48
TN	1.05	0.25	0.95
TP	14.2 **	5.34 *	0.39
AP	0.44	0.37	1.85
TK	5.15 *	7.86 **	3.08
pH	2.02	0.68	1.20
Ca	7.46 *	0.77	2.83
Mg	0.08	3.34	0.87
Richness	16.3 **	0.31	1.86
Shannon index	6.90 *	0.28	1.24
*Acaulospora*	14.3 **	1.84	2.05
*Claroideoglomus*	4.45 *	0.02	0.01
*Diversispora*	6.46 *	0.42	4.81 *
*Gigaspora*	1.02	0.18	0.72
*Glomus*	0.89	1.23	1.23
*Paraglomus*	6.55 *	1.59	2.26
*Redeckera*	<0.01	2.53	0.11
*Scutellospora*	1.17	0.06	0.04

The data indicate the F value Significance levels were marked as follows: * *p* < 0.05 and ** *p* < 0.01. SOC, soil organic carbon; TN, total nitrogen; TP, total phosphorus; AP, available phosphorus; total potassium; Ca, soil exchangeable Ca^2+^; Mg, soil exchangeable Mg^2+^. For the majority of these soil physiochemical properties, as well as AMF diversity and AMF taxa at the genus level, lithology had a more pronounced effect than slope position or their interaction.

## Data Availability

The data is not public, and can be requested from the author if necessary.
